# Unsupervised Stereo Matching with Surface Normal Assistance for Indoor Depth Estimation

**DOI:** 10.3390/s23249850

**Published:** 2023-12-15

**Authors:** Xiule Fan, Ali Jahani Amiri, Baris Fidan, Soo Jeon

**Affiliations:** 1 Department of Mechanical and Mechatronics Engineering, University of Waterloo, 200 University Ave. W., Waterloo, ON N2L 3G1, Canada; x54fan@uwaterloo.ca (X.F.); soojeon@uwaterloo.ca (S.J.); 2 Avidbots Corp., 45 Washburn Dr., Kitchener, ON N2R 1S1, Canada; jahaniam@ualberta.ca

**Keywords:** stereo matching, unsupervised learning, indoor applications, normal estimation

## Abstract

To obtain more accurate depth information with stereo cameras, various learning-based stereo-matching algorithms have been developed recently. These algorithms, however, are significantly affected by textureless regions in indoor applications. To address this problem, we propose a new deep-neural-network-based data-driven stereo-matching scheme that utilizes the surface normal. The proposed scheme includes a neural network and a two-stage training strategy. The neural network involves a feature-extraction module, a normal-estimation branch, and a disparity-estimation branch. The training processes of the feature-extraction module and the normal-estimation branch are supervised while the training of the disparity-estimation branch is performed unsupervised. Experimental results indicate that the proposed scheme is capable of estimating the surface normal accurately in textureless regions, leading to improvement in the disparity-estimation accuracy and stereo-matching quality in indoor applications involving such textureless regions.

## 1. Introduction

Stereo cameras have been widely used by robotic and other intelligent systems to obtain depth information. In such systems, a stereo camera captures a pair of stereo images, from which a stereo-matching algorithm computes the disparity that corresponds to the depth to be estimated. Hence, the accuracy of the stereo-matching algorithm directly affects the quality of the depth estimates.

In the past decades, various stereo-matching algorithms have been proposed. In the early attempts, traditional algorithms [1,2,3,4] were well-studied. Their estimated disparity maps often contain inaccurate or missing estimates. With the help of recent advances in computer hardware technologies as well as developments in deep neural network (DNN) learning, learning-based stereo-matching approaches [5,6,7,8,9] that are trained with large datasets have gained popularity. These data-driven approaches often provide more accurate and denser disparity maps than traditional algorithms do. However, most of these methods are evaluated on either synthetic datasets [10] or outdoor datasets [11,12] collected in driving scenarios.

Estimating depth in an indoor environment using data-driven approaches has been studied previously by adopting various monocular depth-estimation networks trained in a supervised [13,14,15] or unsupervised manner [16,17]. However, existing stereo counterparts are still limited to supervised learning [18] for indoor scenarios. Recently, surface normal has been incorporated into a supervised stereo-based indoor depth-estimation approach [19]. Although supervised approaches may result in high accuracy, obtaining the ground-truth depth labels required for training is a time-consuming and complex process. When the neural network is deployed in an unseen environment, fine tuning with new data is often necessary to maintain its accuracy. The possibility of missing ground-truth information in such new data increases the difficulty of deploying supervised approaches and fine tuning the schemes developed via these approaches. Unsupervised monocular depth-estimation approaches do not rely on expensive ground-truth labels for training; however, they can only estimate depth maps that are up to scale, or otherwise additional information is needed in order to properly scale the estimated depth. The aforementioned challenges can alternatively be addressed by the adoption of an unsupervised stereo-matching approach, where the training does not require ground-truth information and the disparity estimation is not affected by scaling factors.

Unsupervised indoor depth perception is not a trivial task. Compared to outdoor driving scenarios, indoor environments typically consist of more textureless regions. The photometric loss, which is the main supervisory signal in unsupervised monocular and stereo depth estimation, is often ambiguous for these textureless regions [16,17]. Therefore, training the neural network with photometric loss for indoor applications often leads to sub-optimal performance. To reduce the ambiguity due to photometric loss, researchers have attempted to incorporate other information to obtain more reliable supervisory signals. In unsupervised indoor monocular depth estimation, optical flows [16] and superpixels extracted from the input RGB images [17] have been considered. However, this unsupervised strategy is yet to be extended to indoor stereo matching.

In this paper, we study surface normal estimation and its incorporation into unsupervised stereo-matching-based indoor depth estimation. Motivated by the supervised surface-normal-assisted stereo indoor depth-estimation approach that was recently proposed in [19], we design a novel unsupervised scheme consisting of a neural network with three modules and a two-stage training strategy for stereo-depth estimation in indoor environments. The scheme first uses a feature extractor to obtain high-level features from RGB stereo-image inputs and then estimates the surface normal by using the extracted high-level features through its normal-estimation branch. Using the high-level features and the estimated normal maps, the scheme’s disparity-estimation branch generates the disparity estimates. We follow a two-stage strategy to train the DNNs within the proposed scheme in order to achieve unsupervised learning for indoor disparity estimation. First, the feature extractor and normal-estimation-branch DNNs are pre-trained in a supervised manner with the ground-truth surface normal from the NYU v2 dataset [20]. In the second stage after pre-training, we only train the disparity-estimation branch in an unsupervised manner with guidance from the estimated surface normal. The proposed scheme is tested for analysis and performance verification on the NYU v2 dataset for surface normal estimation and on the IRS dataset [18] and InStereo2K dataset [21] for disparity estimation.

The rest of the paper is organized as follows: Section 2 provides a literature review on computer-vision-based indoor depth estimation and the use of stereo matching and surface normal estimation for this purpose. Section 3 describes the overall structure of the neural network in our proposed scheme. Section 4 presents the proposed two-stage training strategy. Section 5 is dedicated to the implementation and evaluation of the proposed scheme using different datasets. The conclusion and final remarks are provided in Section 6.

## 2. Related Work

### 2.1. Surface Normal Estimation

Surface normal estimation has been an important research topic in the computer-vision-research community for more than a decade. In an early attempt, Fouhey et al. [22] designed a support vector machine (SVM) to estimate the surface normal. By grouping pixels according to their geometries and exploiting various cues, the surface normal can also be estimated given an image [23]. The method in [24] combines the information provided by image pixels and segments based on the input images for normal estimation.

Recently, various DNNs have been designed for surface normal estimation. Wang et al. [25] estimated a coarse global normal map and surface normal for image patches and then combined them with a fusion network. Eigen and Fergus [26] developed a multi-scale DNN for multiple computer-vision tasks including surface normal estimation. The surface normal, depth, and information for planar regions predicted by a DNN from an input image are processed by a conditional random field to refine the predictions in [27]. A skip network architecture has also been adopted for normal estimation [28]. GeoNet [29] and its successor GeoNet++ [30] both estimate the depth and surface normal, which are used to refine each other to obtain better estimates. Zhang et al. [31] designed a multi-task network and studied the similarity between estimations at different pixel locations. Such a similarity helps diffuse the surface normal estimates to obtain better results. Liao et al. [32] adopted a spherical regression strategy by using DNN to predict the surface normal. The method introduced in [33] is capable of predicting the normal with a tilted image input. Bae et al. [34] proposed a neural network to first estimate a coarse normal map and its corresponding uncertainty, both of which are combined to form a refined normal map. The encoder–decoder network in [35] learns a discretized representation of high-level features from an input image to support depth estimation and surface normal estimation. Instead of estimating the surface normal from a single image, photometric stereo is another approach that performs normal estimation based on images of the same object in different lighting conditions. Under this formulation, the attention mechanism is used in [36] to estimate a more accurate surface normal of an object with fewer input images. Ju et al. [37] estimated high-resolution normal maps with low-resolution input images.

### 2.2. Stereo Matching

Stereo matching typically consists of four stages [38]: matching cost computation, cost aggregation, disparity computation based on optimization, and disparity refinement. Different traditional stereo-matching algorithms have been proposed by following these steps. These algorithms can be categorized into more efficient local methods [1] and more accurate global methods [2] at the cost of more expensive global optimization. By approximating global optimization in multiple local regions, semi-global methods [3,4] provide a tradeoff between accuracy and efficiency.

The advancement in deep learning has introduced data-driven solutions to the stereo-matching problem, which often lead to better performance. The first attempt in deep stereo matching [39] utilizes a DNN to extract image features, which are then processed by using a traditional method to obtain the estimated disparity. The first end-to-end stereo-matching network was proposed in [5]. Spatial pyramid pooling is adopted in [6] to address ambiguous regions in stereo matching. Redesigning the cost-aggregation module in the neural network also improves the accuracy significantly [7]. Cheng et al. [8] utilized a neural architecture search to identify a design that leads to high-quality results. The attention mechanism and transformer architecture have also been adopted in deep stereo matching [40,41]. Li et al. [42] addressed stereo matching under non-ideal conditions, such as thin structures in the scene and inaccurate image rectification. In addition to accuracy in stereo matching, some other approaches were designed to achieve high-quality estimates in real time by eliminating the stereo-matching cost volume [43] or by performing cost aggregation for inference with 2D convolutions only [44].

Besides supervised stereo matching, research on unsupervised stereo-matching solutions is also popular since they do not depend on additional ground-truth-disparity labels. Unsupervised stereo matching was first studied in [45] by only using confidential regions in the stereo images as inputs. Li and Yuan [46] designed a two-part unsupervised neural network, which estimates an occlusion mask first and then computes disparity in an occlusion-aware manner. Liu et al. [47] explored the use of stereo images captured at different time steps to train their unsupervised neural network. Wang et al. [9] incorporated the recent development in the attention mechanism into their design. A spatially adaptive self-similarity module is introduced in [48] to solve unsupervised stereo matching by using left and right stereo images with different visual properties.

### 2.3. Indoor Depth Estimation

Indoor depth estimation has been studied in both monocular and stereo settings. With the indoor NYU v2 dataset [20], Eigen et al. [13] designed a two-stage neural network to predict a coarse and fine depth map with a monocular RGB input. Other researchers explored different architectures, including conditional random fields [49], random forests [50], adversarial networks [51], and vision transformers (ViT) [15], to improve the estimated depth. Wofk et al. [14] designed a lightweight monocular depth-estimation approach to perform inferences on embedded systems. In addition to the aforementioned supervised monocular methods, unsupervised indoor monocular depth estimation was also studied. Zhou et al. [16] proposed to predict optical flows from temporally consecutive frames captured indoors and use these flows as additional supervisory signals for unsupervised indoor monocular depth estimation. Yu et al. [17] first extracted superpixels from the RGB image and then enforced the planar consistency between the predicted depth map and the superpixels.

In the stereo setting, Kusupati et al. [19] regressed a depth map and surface normal from stereo inputs. Apart from the difference between the ground truth and estimated values, the consistency between the estimated depth and surface normal is also enforced as a training signal. To address the lack of large datasets with stereo images and ground-truth disparity in indoor scenes, a synthetic indoor stereo dataset with 100k frames is proposed in [18]. A smaller but real dataset is also published in [21].

## 3. Proposed Neural Network Design

**Figure 1:**
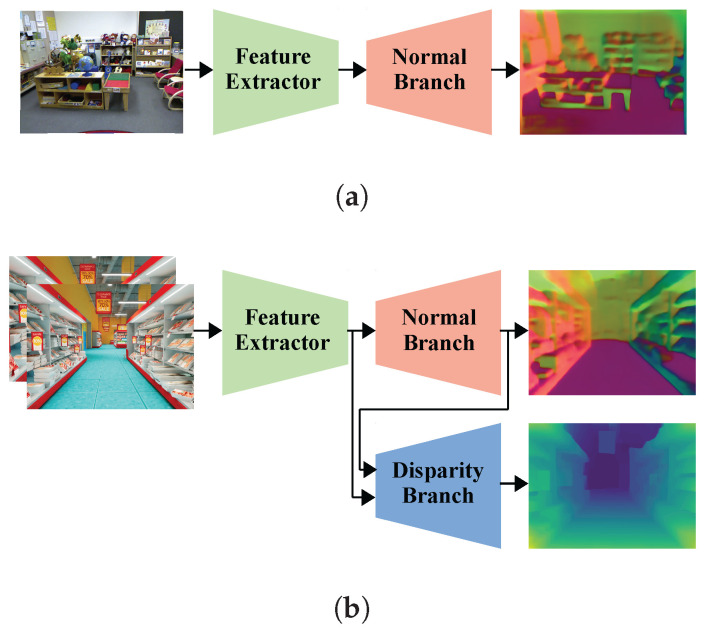
Overview of our proposed approaches for (**a**) normal estimation and (**b**) disparity estimation.

The proposed neural network architecture as shown in Figure 1 consists of three modules: the feature extractor, normal-estimation branch, and disparity-estimation branch. These modules of the proposed scheme can be trained and evaluated in two different modes. In the normal-estimation mode, the feature extractor and normal-estimation branch are used together to produce a surface normal map from an input image. In the disparity-estimation mode, the feature extractor receives stereo images and computes two sets of image features. When training the neural network in the disparity-estimation mode, we use the normal-estimation branch to estimate two surface normal maps by using each set of image features. The disparity-estimation branch then estimates both the left and right disparity maps given the image features and surface normal maps. However, in the evaluation stage, only the left image features are processed by the normal-estimation branch to obtain the left normal map. Using the left and right image features and the left surface normal, the disparity-estimation branch then estimates the left disparity map.

### 3.1. Feature Extraction

**Figure 2:**
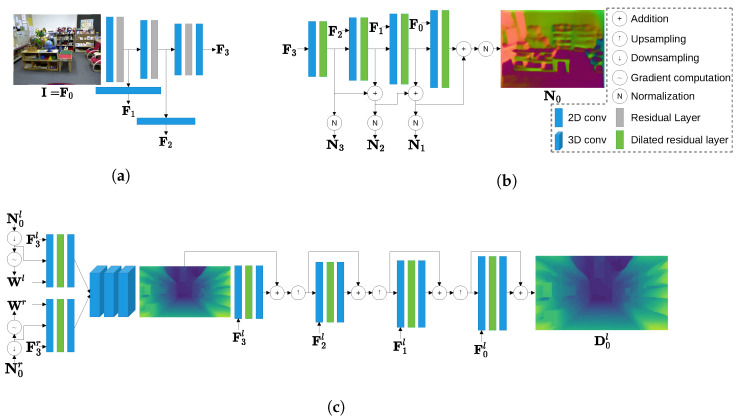
Schematics of different modules in the proposed neural network: (**a**) feature extraction, (**b**) normal-estimation branch, and (**c**) disparity-estimation branch.

The feature extractor is used to downsample the input images and extract a set of high-level features $\left\{F_{0},F_{1},F_{2},F_{3}\right\}$. Its design is inspired by ResNet-50 [52] with three stages, as shown in Figure 2a. We denote the input feature at each stage as $F_{i}^{{}^{\prime }}\in R^{H/2^{i}\times W/2^{i}\times C_{i}}$, where *H* and *W* are the height and width of the input image, respectively; $i\in \left\{0,1,2\right\}$; and $C_{i}$ denotes the number of channels. $F_{i}^{{}^{\prime }}$ is downsampled by a 5×5 convolutional layer with a stride of two, padding of two, batch normalization, and leaky ReLU activation. The output from this layer has half of the spatial resolution compared to $F_{i}^{{}^{\prime }}$ and a higher number of channels $C_{i+1}$. This output is then processed by a series of 3×3 residual layers with leaky ReLU to obtain an intermediate feature $F_{i+1}^{{}^{\prime }}\in R^{H/2^{i+1}\times W/2^{i+1}\times C_{i+1}}$. The output feature $F_{i+1}\in R^{H/2^{i+1}\times W/2^{i+1}\times C_{i+1}}$ from this stage is computed by applying a 3×3 convolution to $F_{i+1}^{{}^{\prime }}$ without an activation function. In the first stage, we set $F_{0}^{{}^{\prime }}=F_{0}=I$, where $I\in R^{H\times W\times 3}$ denotes the input image. $C_{i}$ is set as 3, 32, 64, and 128 for i=0,1,2,3, respectively.

In the normal-estimation mode, we apply this module to one input image I to obtain $\left\{F_{0},F_{1},F_{2},F_{3}\right\}$. In the disparity mode, two sets of image features $\left\{F_{0}^{l},F_{1}^{l},F_{2}^{l},F_{3}^{l}\right\}$ and $\left\{F_{0}^{r},F_{1}^{r},F_{2}^{r},F_{3}^{r}\right\}$ are extracted based on the left and right stereo images, $I^{l}\in R^{H\times W\times 3}$ and $I^{r}\in R^{H\times W\times 3}$, respectively.

### 3.2. Normal-Estimation Branch

After obtaining the high-level image features, we use our proposed modular normal-estimation branch shown in Figure 2b to estimate the surface normal. The normal-estimation branch gradually upsamples the estimated normal maps. Additionally, instead of estimating the surface normal at a higher resolution in each stage, our normal-estimation branch is inspired by a previous stereo-matching network [53] to estimate the surface normal residual at a higher resolution.

At stage *i* of the normal-estimation branch, the image feature $F_{i}$ and an unnormalized surface normal $N_{i+1}^{{}^{\prime }}\in R^{H/2^{i+1}\times W/2^{i+1}\times 3}$ from the previous stage i+1 of this branch are used as the inputs. $N_{i+1}^{{}^{\prime }}$ is first bilinearly upsampled to match the resolution of $F_{i}$ and then concatenated with $F_{i}$ along the channel dimension to form a feature volume. The feature volume is processed by six 3×3 residual blocks with the leaky ReLU activation function while maintaining the same resolution and number of channels. The residual blocks are designed with dilation factors 1, 2, 4, 8, 1, and 1. Next, a 3×3 convolution with no activation functions is applied to the feature volume to compute the surface normal residual $\Delta N_{i}\in R^{H/2^{i}\times W/2^{i}\times 3}$. $\Delta N_{i}$ is then added to the upsampled $N_{i+1}^{{}^{\prime }}$ to compute the unnormalized normal $N_{i}^{{}^{\prime }}\in R^{H/2^{i}\times W/2^{i}\times 3}$. $N_{i}^{{}^{\prime }}$ is used in the next stage of estimation and normalized to $N_{i}\in R^{H/2^{i}\times W/2^{i}\times 3}$ as the output of stage *i*.

There are four stages in the normal-estimation branch in total. To start the normal-estimation process, the upsampling and concatenation steps in stage 3 are neglected. Furthermore, since there is no estimated surface normal at the beginning of this stage, we only use $F_{3}$ as the input and process it with the dilated residual blocks directly. After four stages of computation, the outputs of the normal-estimation branch include $\left\{N_{3},N_{2},N_{1},N_{0}\right\}$. $N_{0}$ is considered the final output of the normal-estimation branch.

### 3.3. Disparity-Estimation Branch

The design of the disparity-estimation branch, as shown in Figure 2c, follows the general architecture adopted by existing data-driven stereo-matching methods [5,6,9,53]. This architecture includes the matching cost construction, cost aggregation, and disparity refinement. To exploit the benefit of the estimated surface normal, we propose an additional normal integration component to combine the surface normal with the matching cost. To introduce our design, we only consider the left stereo view and all estimations derived from this view as examples, unless otherwise stated. The same components can be applied to the right view easily.

#### 3.3.1. Normal Integration

In order to integrate the surface normal information, we treat it as additional features that can be combined with the high-level image features extracted from the feature-extraction module. From the normal-estimation branch, we can obtain the surface normal maps $N_{0}^{l}\in R^{H\times W\times 3}$ and $N_{0}^{r}\in R^{H\times W\times 3}$ for the left and right stereo images, respectively. Using the left view as an example, we first downsample $N_{0}^{l}$ to $N_{0\to 3}^{l}\in R^{H/8\times W/8\times 3}$ with nearest sampling so that its spatial resolution matches that of $F_{3}^{l}$.

From our experiments, we also observe that the estimated surface normal is generally more accurate in regions with smooth estimates than in areas with rapid changes in the surface normal. Integrating inaccurate surface normal information into the matching cost may introduce negative effects in stereo matching. Therefore, it is important that the neural network focuses on accurate normal estimates and ignores the inaccurate ones. To achieve this goal, we propose a weighting mask based on our observation. This weighting mask places higher weights at smooth regions and lower weights when the surface normal changes significantly. In image processing, the Laplacian filter is commonly used to capture edges or intensity changes, which means it can also be used to identify image patches with minimal variations. By using this filter, the weighting mask that we design is:

(1) $$W^{l}=\exp\left(-\lambda_{w}\sum_{j=1}^{3}\left|\nabla^{2}N_{0\to 3}^{l}\left(\cdot ,\cdot ,j\right)\right|\right)\in R^{H\times W},$$

where $\lambda_{w}=5$ is a constant to control the sensitivity and $\nabla^{2}$ denotes a 3×3 Laplacian filter. The resulting values from the Laplacian filters have lower magnitudes at regions with a smoother surface normal. To remove the ambiguity introduced by signs, we consider the absolute value of these resulting features. Then, we perform summation along the channel dimension to combine the surface normal smoothness in different directions. Lastly, the exponential function constrains the weighting mask to be between 0 and 1.

After obtaining the downsampled estimated surface normal and weighting mask, we concatenate $F_{3}^{l}$, $N_{0\to 3}^{l}$, and $W^{l}$ along the channel dimension and process this volume by a 3×3 convolution followed by batch normalization and leaky ReLU activation to change its number of channels to 256. Then, we apply dilated residual blocks, which follow the same design as introduced in Section 3.2, to balance the values in the combined feature while maintaining the same spatial resolution and number of channels. Lastly, another 3×3 convolution without batch normalization or an activation function computes the output volume $F_{3}^{{}^{\prime }l}\in R^{H/8\times W/8\times 256}$ from this component. This volume contains both information obtained directly from the input image and the estimated surface normal. Building a stereo-matching cost with this volume allows us to take advantage of accurate normal estimates.

#### 3.3.2. Matching Cost Construction

From the left and right combined features $F_{3}^{{}^{\prime }l}$ and $F_{3}^{{}^{\prime }r}$, we construct a stereo-matching cost volume by considering one of them as the reference feature, while the other feature is considered the target feature. The difference between the reference feature and the target feature that shifted according to all disparity candidates is computed as the cost volume [53]. If we assume that the number of disparity candidates at the original image resolution is *D*, there are d=D/8 candidates at the lowest image resolution. When using $F_{3}^{{}^{\prime }l}$ as the reference feature, we obtain a left matching cost $C^{l}\in R^{H/8\times W/8\times 256\times d}$.

#### 3.3.3. Cost Aggregation

To enable more robust stereo matching, we perform cost aggregation on the matching costs. Cost aggregation in a data-driven stereo-matching approach is achieved by applying 3D convolutions to the cost volume along the spatial and disparity dimensions [5,6,53]. We follow [53] to design a lightweight cost-aggregation module with five 3D 3×3×3 convolutional layers. The first four 3D convolutions are followed by batch normalization and leaky ReLU activation. They also maintain the number of channels for the cost volume at 256. The last convolution reduces the channel number to one to obtain an aggregated cost, from which a left initial disparity $D_{init}^{l}\in R^{H/8\times W/8}$ is regressed through the differentiable soft argmin introduced in [5].

#### 3.3.4. Disparity Refinement

Although the cost-aggregation module can compute an initial disparity map, $D_{init}^{l}$ may not include detailed estimates. To remedy this problem, we design a disparity-refinement module to gradually upsample $D_{init}^{l}$ while introducing more details. Similar to the normal-estimation branch, the refinement module adopts a modular design with multiple stages.

The inputs of stage *i* include the refined disparity from the previous refinement stage $D_{i+1}^{l}\in R^{H/2^{i+1}\times W/2^{i+1}}$ and the left high-level feature $F_{i}^{l}$, while its output is the refined disparity map at a higher resolution $D_{i}^{l}\in R^{H/2^{i}\times W/2^{i}}$. In this refinement stage, $D_{i+1}^{l}$ is first bilinearly upsampled to match the resolution of $F_{i}^{l}$. The upsampled disparity and $F_{i}^{l}$ are then concatenated and processed by a 3×3 convolution without batch normalization or activation functions to reduce its channel number to 32. Dilated residual blocks as described in Section 3.2 are also applied to this volume. Following the residual blocks, the volume undergoes another 3×3 convolution with no batch normalization or activation functions, resulting in a disparity residual. The disparity residual is added to the upsampled disparity. After addition, this refined disparity map passes through a ReLU activation function to obtain a $D_{i}^{l}$ whose values are all non-negative.

Similar to the normal-estimation branch, the refinement module also includes four stages. At the first stage of refinement, which is stage 3, the upsampling step is neglected and the upsampled disparity is replaced by $D_{init}^{l}$. $D_{0}^{l}$ at the original image resolution is used as the final output of the disparity-estimation branch.

## 4. Training Strategy

### 4.1. Training for Normal Estimation

In the normal mode, the neural network is trained in a supervised manner. The supervised learning of surface normal estimation commonly relies on either the cosine similarity loss [26,37] or the L2 loss [30,33,36]. We adopt the latter alternative since it yields a better performance. With the set of estimated surface normal maps $\left\{N_{3},N_{2},N_{1},N_{0}\right\}$ from an input image, the supervised loss is

(2) $$L_{n}=\sum_{i=0}^{3}\left(\frac{1}{2^{i}HW}\sum_{p}{∥N_{i\to 0}\left(p\right)-N^{*}\left(p\right)∥}_{2}\right),$$

where $N_{i\to 0}$ denotes the estimated surface normal $N_{i}$ bilinearly upsampled to the same resolution as the input image, $N^{*}$ denotes the ground-truth normal, and p denotes an arbitrary pixel. The weighting term $1/2^{i}$ enforces the training loss to focus on estimates at higher image resolutions. Note that only the feature extractor and normal-estimation branch are utilized to estimate the surface normal. Hence, only the parameters in these two modules are updated with (2).

### 4.2. Training for Disparity Estimation

After the neural network obtains preliminary knowledge on surface normal estimation, we further train it for disparity estimation in a fully unsupervised manner. In this stage of training, the parameters of the feature extractor and surface normal-estimation branch are frozen. Therefore, back propagation is only allowed in the disparity-estimation branch. This training stage involves multiple training losses whose definitions are given below by using the left view as an example. By applying a similar formulation, these losses can be expanded to the right view.

#### 4.2.1. Photometric Loss

The photometric loss quantifies the differences between one stereo image and a reconstructed image based on the other stereo view and disparity. If the disparity is accurate, the stereo image and the reconstructed view are visually similar. Hence, the photometric loss will be close to zero. The photometric loss of a left-view pixel is defined as

(3) $$L_{ph,i}^{l}\left(p\right)=\frac{\alpha }{2}\left(1-SSIM\left(I^{l}\left(p\right),{\hat{I}}_{i}^{l}\left(p\right)\right)\right)+\left(1-\alpha \right)∥I^{l}\left(p\right)-{\hat{I}}_{i}^{l}\left(p\right)∥,$$

where α=0.85 and $SSIM\left(\cdot \right)$ denotes the structural similarity index measure [54]. ${\hat{I}}_{i}^{l}\in R^{H\times W\times 3}$ is a bilinearly reconstructed image according to the right stereo view $I^{r}$ and a disparity map $D_{i\to 0}^{l}\in R^{H\times W}$, which is bilinearly upsampled from the estimated left disparity map $D_{i}^{l}$ at refinement stage *i*.

#### 4.2.2. Disparity Smoothness Loss

To prevent the neural network from estimating noisy disparity maps, a disparity smoothness loss is widely used to regularize the estimates. This smoothness loss is given as

(4) $$\begin{array}{c} L_{ds,i}^{l}\left(p\right)=\left|\nabla_{x}D_{i\to 0}^{l}\left(p\right)\right|e^{-∥\nabla_{x}I^{l}\left(p\right)∥}+\left|\nabla_{y}D_{i\to 0}^{l}\left(p\right)\right|e^{-∥\nabla_{y}I^{l}\left(p\right)∥}, \end{array}$$

where $\nabla_{x}$ and $\nabla_{y}$ are the gradients of an image with respect to the horizontal and vertical direction, respectively. The gradients in (4) emphasize disparity smoothness at textureless regions since these regions are more likely to exhibit smooth disparity.

#### 4.2.3. Normal Consistency Loss

In addition to the photometric and disparity smoothness losses, we further exploit the consistency between the estimated normal and disparity to improve estimation at ambiguous regions. The normal consistency loss is defined as

(5) $$L_{n,i}^{l}\left(p\right)=W_{i\to 0}^{l}\left(p\right){∥N_{i\to 0}^{l}\left(p\right)-N_{D,i\to 0}^{l}\left(p\right)∥}_{2},$$

where $N_{D,i\to 0}^{l}\in R^{H\times W\times 3}$ denotes the surface normal converted from the upsampled disparity map $D_{i\to 0}^{l}$ according to [18], and the weight $W_{i\to 0}^{l}\in R^{H\times W}$ is obtained by applying (1) to the upsampled left estimated surface normal map $N_{i\to 0}^{l}$. The weight can constrain the normal consistency loss at smoother regions, which usually contain more accurate normal estimates.

#### 4.2.4. Left–Right Consistency Loss

To address occlusion, which is a common problem in stereo matching, a left–right consistency loss is used. This loss is given as

(6) $$L_{lr,i}^{l}\left(p\right)=\left|D_{i\to 0}^{l}\left(p\right)-{\hat{D}}_{i\to 0}^{l}\left(p\right)\right|,$$

where ${\hat{D}}_{i\to 0}^{l}\in R^{H\times W}$ is a reconstructed left disparity map by bilinearly sampling the upsampled right disparity map $D_{i\to 0}^{r}$ according to the upsampled left disparity map $D_{i\to 0}^{l}$.

Moreover, since our network can estimate multi-scale disparity and normal maps, we utilize estimates at all scales to train the disparity-estimation branch. The combined training loss based on left and right estimates at scale *i* is

(7) $$\begin{array}{cc} \; & L_{d,i}=\sum_{p}\alpha_{ph}\left(L_{ph,i}^{l}\left(p\right)+L_{ph,i}^{r}\left(p\right)\right)+\alpha_{ds}\left(L_{ds,i}^{l}\left(p\right)+L_{ds,i}^{r}\left(p\right)\right)\\ & +\alpha_{n}\left(L_{n,i}^{l}\left(p\right)+L_{n,i}^{r}\left(p\right)\right)+\alpha_{lr}\left(L_{lr,i}^{l}\left(p\right)+L_{lr,i}^{r}\left(p\right)\right), \end{array}$$

where the superscript *r* denotes that the losses are based on the right-view images, and the α’s are the weights for different terms. By collecting the training losses at all scales, the final loss for disparity training is

(8) $$L_{d}=\frac{1}{4HW}\sum_{i=0}^{3}\left(\frac{1}{2^{i}}L_{d,i}\right).$$

## 5. Experimental Results

### 5.1. Implementation Details

We train and evaluate our proposed scheme on multiple datasets for normal and disparity estimations. For normal estimation, we apply our design to the NYU v2 dataset [20]. The availability of large public datasets with indoor stereo images and ground-truth disparity is limited. Therefore, we train our network by using the large synthetic IRS dataset [18] for indoor stereo matching. The IRS dataset consists of images rendered in both bright and dark lighting conditions. Since low-light scenarios are out of the scope of this study, we only include images rendered in normal lighting in training and evaluation. To evaluate our method’s generalization ability, we further test it with a smaller real indoor dataset, InStereo2K [21].

In both training stages, the neural network is trained by using an Adam optimizer. Data augmentation is applied to all training images by randomly modifying their brightness, contrast, saturation, and hue. All images are normalized by the ImageNet mean and variance. During training for normal estimation, the images are randomly cropped to a resolution of 416×552. The neural network is then trained by using data from the NYU v2 dataset with a batch size of eight for 20 epochs. The initial learning rate in the first stage is 0.001. This learning rate is later reduced by half at the 10th epoch. After training for normal estimation is completed, the disparity-estimation branch is fine tuned on the IRS dataset for another 20 epochs with a batch size of four. The initial learning rate is 0.0001, which is multiplied by 0.1 at the 10th epoch. The input images are randomly cropped to a resolution of 256×512. The constants chosen for (7) are $\alpha_{ph}=5$, $\alpha_{ds}=0.05$, $\alpha_{n}=0.5$, and $\alpha_{lr}=0.01$. The negative slope of all leaky ReLU activation functions is chosen as 0.2.

### 5.2. NYU v2 Dataset

We compare the performance of our approach on normal estimation with existing methods on the NYU v2 [20] test set. We report the performance by using error and accuracy metrics, both of which are based on the angular difference between the estimated and ground-truth normal vectors. The error metrics include the mean error, median error, and root mean squared error (RMSE) of the angular differences at all pixel locations. The accuracy metrics are the percentages of pixels with angular differences lower than $11.25^{\circ }$, $22.5^{\circ }$, and $30^{\circ }$, respectively.

The quantitative results are summarized in Table 1. Although the main focus of our work is indoor stereo matching instead of surface normal estimation, our feature extractor and normal-estimation branch can still compute accurate surface normal estimates. Compared to the majority of the existing methods in Table 1, our approach achieves a lower error and higher accuracy. The performance of our method only falls behind that of [34,35] even though we did not specifically tune our neural network or utilize an intricate design tailored for surface normal estimation. Although it is possible to utilize the surface normal estimated from [34,35] to guide the downstream disparity-estimation process, this approach will significantly increase the complexity. For instance, we can no longer use the same feature-extraction module for both tasks, which implies a possible higher memory footprint and computational power requirement to train and use the entire architecture. Moreover, the prediction step in [34] relies on both the estimated normal and an uncertainty map, which introduces additional complexity compared to our approach. In [35], an extra internal discretization module is needed in addition to the regular encoder–decoder design. On the other hand, our method offers a simplistic solution to surface normal estimation while maintaining high accuracy. Since the main goal of our proposed scheme is disparity estimation and surface normal estimation only serves as a support role to our main goal, it is important to keep the surface-normal-estimation solution simple to avoid adding unnecessary overhead to the overall scheme.

**Figure 3:**
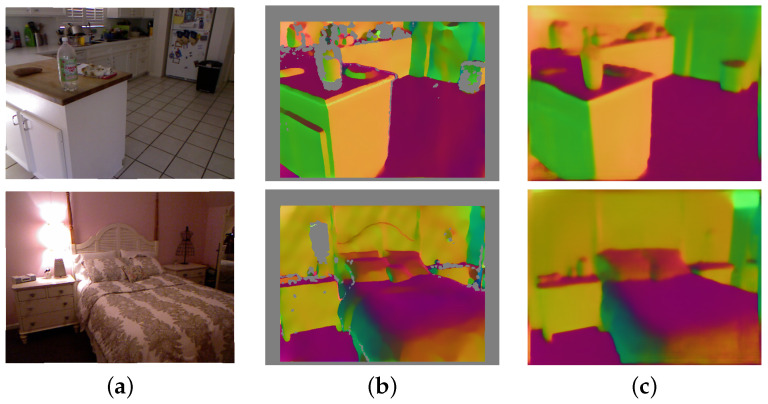
Sample qualitative results of surface normal estimation by our approach using the NYU v2 test set: (**a**) input RGB images, (**b**) ground-truth surface normal, (**c**) estimated surface normal.

In addition to the quantitative comparison, we present some qualitative results obtained by our approach in Figure 3. According to these results, our method can compute high-quality surface normal estimates, especially at smooth and often textureless regions that are commonly seen in indoor environments. Examples of these regions can be found in Figure 3 in the top image on the counter and in the bottom image on the wall and floor areas. These regions often lead to ambiguous results in unsupervised stereo matching. This observation suggests that our estimated normal may contain useful information to address ambiguity in unsupervised indoor disparity estimation.

**Figure 4:**
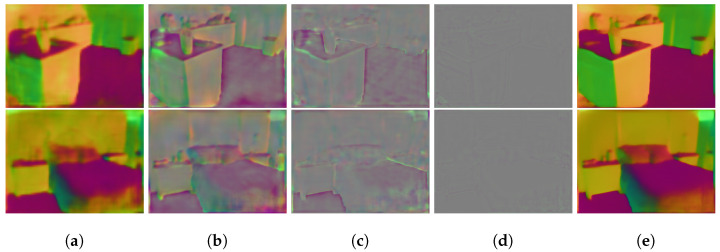
Visualization of the estimated normal residuals from the normal-estimation branch: (**a**) initial estimated surface normal at stage 3 of the normal-estimation branch, (**b**–**d**): surface normal residuals obtained at stage 2 to 0, (**e**) final estimated normal.

One unique design of our normal-estimation branch is its ability to estimate surface normal residuals to refine normal estimates from the previous stage. We provide visualization in Figure 4 to demonstrate these residuals. It can be seen that the surface normal residuals recover a substantial amount of missing information at an earlier stage (e.g., stage 2) of the normal-estimation branch, especially at large flat regions. At stage 0, which is close to the end of the network, the residuals only need to correct the normal estimates at object boundaries.

### 5.3. IRS Dataset

The evaluation of disparity estimation is first performed on the IRS dataset’s [18] test set after training the neural network for stereo matching. Since indoor stereo matching is a less-explored topic, existing work on this topic is limited. Based on the availability of existing work and open-source code, we select FADNet [18], GwcNet [56], and PASMnet [9] for comparison. The first two approaches are supervised methods, while the last one is based on unsupervised training. The quantitative comparison is outlined in Table 2 based on two metrics: the endpoint error (EPE) and percentage of pixels with an error of more than 3 px (>3 px). The former metric quantifies the error, while the latter quantifies the accuracy of different approaches. Furthermore, we compute these two metrics in two scenarios: using all the pixels in the images (EPE-a and >3 px-a) and using textureless pixels in the images (EPE-t and >3 px-t). To extract the textureless pixels, we first apply an 11×11 Laplacian filter to the input RGB images. After calculating the absolute value and summation across the channel dimension, we label a pixel as textureless if its resulting value is less than or equal to one.

Among all four methods included in Table 2, the supervised FADNet [18] achieves the lowest EPE. Even though our approach is an unsupervised method, it still outperforms another supervised method [56] with a lower error. Compared to the recent open-source unsupervised stereo-matching approach [9], our method estimates disparity with a lower EPE and fewer outliers (>3 px). Our approach results in a decrease in the EPE and in >3 px by 0.69% and 4.97%, respectively, when all the pixels are considered in comparison with [9]. At textureless regions, the EPE and >3 px are lower by 1.68% and 3.54%, respectively. These results demonstrate that our approach is effective at estimating more accurate disparity at textureless regions, especially in terms of the EPE.

**Figure 5:**
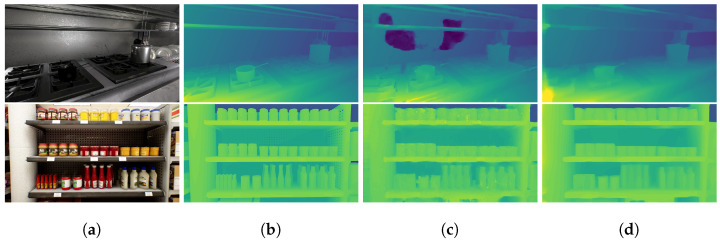
Sample qualitative results of disparity estimation on the IRS dataset test set: (**a**) input RGB images, (**b**) ground-truth disparity, (**c**) estimated disparity using [9], and (**d**) estimated disparity from our method.

Apart from the quantitative results, we present sample qualitative results in Figure 5. The qualitative results demonstrate that the estimated disparity using our method is significantly better at planar and textureless regions than the estimates from [9]. This observation is supported by the back of the stove shown in the top image of Figure 5. In this example, PASMnet fails to understand that the gray wall at the back is a planar region and computes holes in the estimated disparity map. Our method successfully estimates a smooth disparity transition in that area to represent a plane.

### 5.4. InStereo2K Dataset

To further evaluate the performance of our approach in indoor stereo matching, we further study its generalization ability. This study is completed by performing inference directly on the InStereo2K [21] test set by using our method and [9], both of which are only trained on the IRS dataset for stereo matching. The quantitative results are shown in Table 3. It can be seen that our method outperforms [9] with a lower error and percentage of outliers using both all pixels and textureless pixels. The results indicate that our approach improves the EPE and >3 px by 7.35% and 14.23%, respectively, when using all the pixels, as well as 5.90% and 13.04% in the EPE and >3 px, respectively, when considering the textureless pixels. These results demonstrate the better generalization ability of our approach.

**Figure 6:**
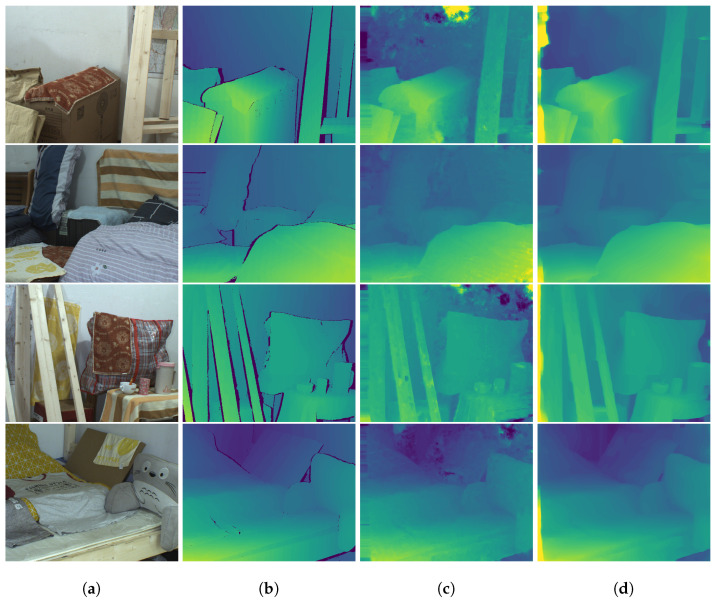
Sample qualitative results of disparity estimation on the InStereo2K test set: (**a**) input RGB images, (**b**) ground-truth disparity, (**c**) estimated disparity using [9], and (**d**) estimated disparity using our method.

The qualitative results from Figure 6 show that both methods have difficulties estimating accurate disparity at the leftmost occluded areas, which are generally challenging to estimate correctly. However, the estimates at textureless and planar regions using our method are smoother and more accurate. These estimates can be found in the wall areas in the first and third row from the top in Figure 6. Additionally, our approach also captures object boundaries more clearly, which can be seen at the wood sticks and pillows in Figure 6.

In addition to the evaluation of accuracy, we performed a time study on both methods by using the same dataset. The results are shown in Table 3. Our approach can process the images at an average rate of 14.01 frames per second (FPS) on an NVIDIA RTX 3060 GPU, which is significantly faster than the 7.20 FPS achieved by the PASMnet.

### 5.5. Ablation Study

Based on the previous experimental results, it can be seen that our proposed unsupervised stereo-matching scheme is effective in indoor stereo matching. To further understand how the surface normal contributes to our problem, we study the effectiveness of each design component related to normal estimation. In our proposed scheme, surface normal information is incorporated through three main design components: pre-training the feature extractor and normal-estimation branch for the normal-estimation task, normal consistency loss in (5), and the normal integration component introduced in Section 3.3.1. In this ablation study, we design four different configurations based on our proposed scheme by disabling and enabling some of these design components.

In our baseline configuration (Configuration I), the neural network only consists of the feature extractor and the disparity-estimation branch without the normal integration component. Additionally, the feature extractor has not been pre-trained on the NYU v2 dataset. Unsupervised training of this configuration for disparity estimation only relies on (3), (4), and (6). This configuration represents an unsupervised stereo-matching scheme without any surface normal information. Building upon Configuration I, we introduce the normal-estimation branch in Configuration II. Additionally, both the feature extractor and normal-estimation branch are pre-trained with the NYU v2 dataset in this configuration. In Configuration III, we further include the normal consistency loss (5) in the unsupervised training process for disparity estimation. Lastly, the normal integration component is incorporated into the disparity-estimation branch in Configuration IV. Configuration IV also represents our proposed scheme. From Configuration I to IV, more and more surface normal information is included. Comparing these configurations can demonstrate that each additional piece of normal information is beneficial to unsupervised indoor stereo matching.

**Figure 7:**
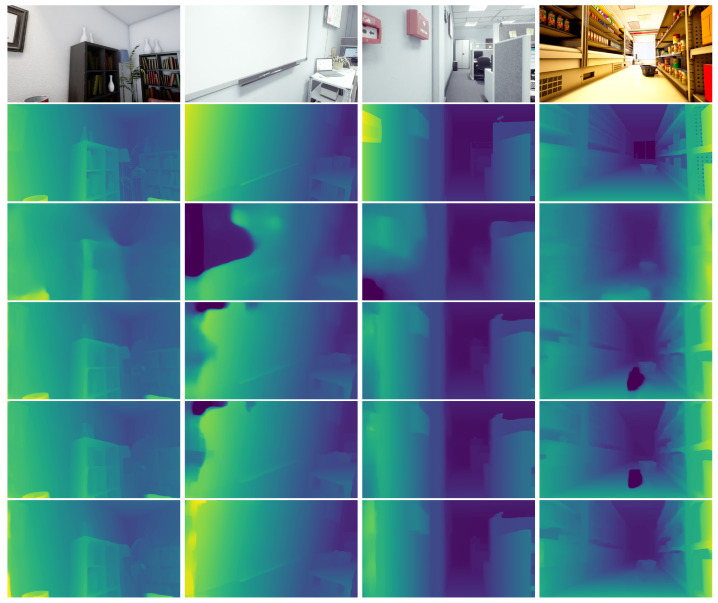
Sample qualitative results from the ablation study. The images from top to bottom are RGB image inputs, ground-truth disparity, and disparity estimates using Configurations I to IV as described in Table 4.

The results of the ablation study are summarized in Table 4 and Figure 7. According to the quantitative results in Table 4, Configuration I estimates disparity with a significantly higher error and more outliers compared to the other configurations. Pre-training the feature extractor in Configuration II improves the disparity estimation considerably by a 41.02% decrease in the EPE and a 45.55% decrease in >3 px. Configuration II mainly relies on the photometric loss as the main supervisory signal. Introducing (5) into the training loss results in a lower EPE and >3 px by 3.41% and 5.49%, respectively. Configuration IV further incorporates the normal integration component, which leads to the most accurate disparity estimates among all four configurations with a 0.31% and 2.12% decrease in the EPE and >3 px, respectively, compared to Configuration III. This configuration is also the one we adopt as our final design.

From the qualitative results in Figure 7, we can see that the estimated disparity maps from Configuration I are blurry with many inaccurate disparity estimates, especially at textureless regions, such as the wall, whiteboard, and floor. After pre-training the neural network with the NYU v2 dataset in Configuration II, more-defined object boundaries are captured at the shelf and table areas. However, significant errors are still visible at objects with low textures. As the normal consistency loss and normal integration component are included, the quality of the estimated disparity maps increases, especially at large, flat, and textureless regions that are typically ambiguous for stereo matching. This can be observed in the disparity maps computed by Configuration IV. These disparity maps contain smooth and accurate disparity estimates at textureless areas.

Overall, the above results demonstrate the significance of integrating surface normal information into unsupervised indoor stereo matching. Since pre-training the network for surface normal estimation, the normal consistency loss (5), and the normal integration component introduced in Section 3.3.1 involve standalone designs independent of other modules and training losses typically used in deep-learning-based stereo matching, we expect them to provide a similar performance improvement when they are integrated with supervised stereo-matching approaches for indoor applications. However, further experiments are required to formally demonstrate this.

## 6. Conclusions

In this work, we addressed the problem of unsupervised indoor stereo matching. We proposed a neural network design that consists of a feature extractor, a surface normal-estimation branch, and a disparity-estimation branch. The training of our network is performed in two stages. First, the extraction module and the normal-estimation branch are trained to estimate the surface normal with supervised learning by using the NYU v2 dataset. The disparity-estimation branch is then trained in an unsupervised manner while incorporating the surface normal estimated by the normal-estimation branch. Due to the lack of large datasets with real indoor stereo images, the second stage of training is carried out by using a large synthetic indoor stereo dataset. Experimental results demonstrate that the normal-estimation branch estimates the surface normal accurately. With the aid of normal estimation, the disparity-estimation branch estimates high-quality disparity for indoor scenes. Our method achieves higher accuracy in disparity estimation than a recent unsupervised method. It also demonstrates a better generalization ability when it is applied to images that are visually different from the training images.

As a future direction, the proposed design may be further refined by jointly improving the normal-estimation branch and the disparity-estimation branch. Unsupervised surface normal estimation may be approached to reach a fully unsupervised training strategy. It is also important to quantify the effectiveness of integrating surface normal information into a supervised stereo-matching method to further understand its potential for indoor scenarios. Lastly, integrating this method with a robotic system for various applications is another future direction of study.

## Figures and Tables

**Table 1:** Error and accuracy metrics of different surface-normal-estimation methods on the NYU v2 test set.

Method	Error $\left({}^{\circ }\right)$			Accuracy (%)		
	**Mean**	**Median**	**RMSE**	$11.25^{\circ }$	$22.5^{\circ }$	$30^{\circ }$
Wang et al. [25]	26.9	14.8	-	42.0	61.2	68.2
Multi-scale [26]	20.9	13.2	-	44.4	67.2	75.9
SURGE [27]	20.6	12.2	-	47.3	68.9	76.6
Bansal et al. [28]	19.8	12.0	28.2	47.9	70.0	77.8
GeoNet [29]	19.0	11.8	26.9	48.4	71.5	79.5
Nekrasov et al. [55]	24.0	17.7	-	-	-	-
PAP [31]	18.6	11.7	25.5	48.8	72.2	79.8
Liao et al. [32]	19.7	12.5	-	45.8	72.1	80.6
Bae et al. [34]	14.9	7.5	23.5	62.2	79.3	85.2
GeoNet++ [30]	18.5	11.2	26.7	50.2	73.2	80.7
iDisc [35]	14.6	7.3	22.8	63.8	79.8	85.6
Ours	17.8	11.3	25.4	52.1	74.2	81.6

**Table 2:** Quantitative comparison for disparity estimation on the test set of the IRS dataset in terms of error and accuracy. Different methods are separated into supervised (Sup.) methods and unsupervised (Unsup.) ones.

Method	Training	EPE-a (px)	>3 px-a (%)	EPE-t (px)	>3 px-t (%)
FADNet [18]	Sup.	0.75	-	-	-
GwcNet [56]	Sup.	3.01	-	-	-
PASMnet [9]	Unsup.	2.91	15.08	2.97	14.96
Ours	Unsup.	2.89	14.33	2.92	14.43

**Table 3:** Quantitative results for disparity estimation on InStereo2K test set.

Method	EPE-a (px)	>3 px-a (%)	EPE-t (px)	>3 px-t (%)	FPS
PASMnet [9]	3.13	15.18	3.90	19.32	7.20
Ours	2.90	13.02	3.67	16.80	14.01

**Table 4:** Quantitative results from the ablation study for disparity estimation with different configurations.

Configuration	Pre-Train	$L_{n}$	Normal Integration	EPE-a (px)	>3 px-a (%)
I				5.080	28.45
II	✓			2.996	15.49
III	✓	✓		2.894	14.64
IV	✓	✓	✓	2.885	14.33

## Data Availability

No new data were created or analyzed in this study. Data sharing is not applicable to this article.
